# Abrupt onset or exacerbation of anorexia nervosa following recent infections: a mini-review and a case report with an atypical manifestation of PANS

**DOI:** 10.1007/s40519-025-01721-8

**Published:** 2025-02-06

**Authors:** Lavalle Raffaele, Tournour Cristina, Rubano Serena, Delsedime Nadia, Panero Matteo, Abbate-Daga Giovanni

**Affiliations:** Department of Neuroscience “Rita Levi Montalcini”, University of Turin, Turin, Italy

**Keywords:** Anorexia nervosa, Food restrictions, PANDAS, PANS, Abrupt onset, Post-infectious onset

## Abstract

**Purpose:**

This study examines the connection between infections and the abrupt onset or exacerbation of anorexia nervosa (AN) in the context of PANS (Pediatric Acute–Onset Neuropsychiatric Syndrome) and PANDAS (Pediatric Autoimmune Neuropsychiatric Disorders Associated with Streptococcal infections).

**Methods:**

The authors conducted a literature review and present a case study of a 17-year-old girl treated at the University of Turin Eating Disorder Unit, whose AN presented with an abrupt onset following an infection and was characterized by a rapid and favorable course.

**Results:**

The review includes 30 cases derived from six studies, suggesting that AN can manifest as PANS/PANDAS, with subjects, mainly adolescents, experiencing sudden or exacerbated eating restrictions alongside obsessive–compulsive behaviors. Some cases improved with antibiotics or standard psychiatric treatments, although age, symptom severity, and diagnostic markers like D8/17 varied across studies. Data on treatment follow-up also varied.

The report refers to the case of V., a 17-year-old girl who developed AN following an intestinal infection. After significant weight loss and amenorrhea, she was hospitalized for 29 days, receiving psychiatric care, nutritional rehabilitation, and pharmacological treatment. Over 2 months of residential care and ongoing outpatient therapy, she improved notably, though mild body dysmorphophobia persisted.

**Conclusions:**

While evidence suggests a possible link between infections and AN, research is still limited and inconsistent. Infections may trigger AN through autoimmune mechanisms or by initiating weight loss, particularly in younger patients. Although further studies are needed to clarify this relationship, infections should be considered in AN diagnosis, especially in pediatric cases.

**Level of Evidence:** Level V, a narrative review and a case report.

## Introduction

The pediatric acute-onset neuropsychiatric syndrome (PANS) has been described as a peculiar clinical presentation, where a sudden beginning of severe food restrictions or obsessive–compulsive disorder co-occurs with further neuropsychiatric symptoms not better justified by a medical disorder [1]. Instead, the construct of “Pediatric autoimmune neuropsychiatric disorders associated with streptococcal infections” (PANDAS) [2] more specifically refers to those cases of abrupt prepubertal onset of “Obsessive–Compulsive Disorder” (OCD) or tics prompted by a precedent Group A Streptococcus, GAS, infection (Table 1). Before the term “PANS” was coined, some authors [3] introduced the concept of PANDAS–AN, defined as a particular syndrome characterized as a prepubertal, sudden onset or exacerbation of AN, with evidence of antecedent or concomitant streptococcal infection (positivity to throat culture and/or serological findings, anti-DNase B or ASO titers), further psychiatric or neurological abnormalities not occurring exclusively during stress or another medical condition. However, the overlap between the criteria for PANS, specifically its subset with eating restrictions, and the criteria for PANDAS–AN appears to be only partial. In fact, eating restrictions, as mentioned in the first criterion for PANS, are not sufficient to establish a full diagnosis of AN (restriction of caloric intake leading to a significantly low body weight, intense fear of gaining weight, and body image disturbance [4]). The onset of AN generally occurs in adolescence [5] and is marked by core psychopathological features, including obsessive thoughts about food as well as significant anxiety or depressive symptoms related to it [6–8].

**Table 1:** PANS and PANDAS criteria

PANS	PANDAS
I. Acute onset of OCD or severely restricted food intake II. Concomitant additional neuropsychiatric symptoms, at least two, with a similarly abrupt onset (anxiety, depression, irritability, etc.) III. Symptoms are not better justified by a known neurologic or medical disorder	I. OCD/tics II. Age Requirement (from 3 years to puberty) III. Abrupt onset and episodic course IV. Association with Group A Streptococcal infection V. Neurological Abnormalities

The nosological construct of PANS considers both eating disorder and OCD psychiatric diagnoses as alternative possibilities to fulfill the first diagnostic criterion. Thus, the introduction of the PANS/PANDAS diagnostic category has empowered a new reconceptualization of psychiatric illnesses potentially caused by an autoimmune process triggered by a recent infection. Some evidence of an association between mental disorders and immune mechanisms has been produced: for example, a large Danish epidemiological study on 1,067,743 children < 18 years of age demonstrated an association between mental disorders and previous infections [9]. Furthermore, some researchers hypothesize a complex possible link between autoimmunity and AN [10].

However, other authors questioned the validity of the construct of PANDAS–AN, highlighting several methodological limitations, such as the low specificity in the diagnostic tests, the apparent low prevalence, the dubious specificity of the association between type of infection and specific diagnosis of mental disorder and the uncertainty in diagnostic criteria [11]. As Hyman [12] said, the better conclusion is that the nexus of infection, immunology, and mental illness has been inadequately described and explored.

In the present study, we present a mini-review of all the cases of abrupt onset or exacerbation of AN or eating restrictions—subsequent to a previous infection (PANS/PANDAS-AN)—we were able to retrieve in the scientific literature. Furthermore, we also describe the case of a young girl who accessed our Eating Disorder Unit (University of Turin—AOU Città della Salute Hospital) for the specificity of its onset, the clinical characteristics, and the rapid and favorable course. In summary, the aim is to stimulate the discussion on the topic through (a) a synthesis of the past literature on the theme, (b) the description of an atypical case in adolescence close to adulthood.

## Methods

Online databases, including PubMed, Embase, and PsycInfo, were searched by the authors. Subsequently, the reference lists of all included studies were scanned to detect possible further papers meeting the inclusion criteria. The following inclusion criteria were employed: (a) studies or case reports/series focusing on subjects with abrupt onset or exacerbation of AN or eating restrictions subsequent to a previous infection, not secondary to other mental illnesses (such as OCD), thus individuals suffering from PANS or PANDAS–AN; (b) original research articles; and (c) English, Italian, Spanish or French language articles. No time filters were applied until 15 May 2024. We included the following search keywords: “anorexia nervosa” AND (“PANS” OR “PANDAS”).

## Mini-review of the cases available in literature

At present, only a few cases with characteristics suggestive of PANS/PANDAS with an acute onset or exacerbation of AN or eating restrictions have been described in the literature: cases mostly come from a reduced number of study groups, most of them are around the age of puberty, and there is a lack of recently published case reports. We computed the weighted mean age of onset or exacerbation of symptoms of the cases included in the present review, which results in 13.86 years, and the weighted mean age of subjects at the moment of assessment, which amounts to 15.62 years. However, these mean values were not entirely reliable, as the age of onset or exacerbation ranged from 6 years and 11 months to 19 years, and age at the moment of assessment ranged from 7 to 35 years. Therefore, due to this significant variability in age values, we also calculated the median values of the subjects included in the present work, which turned out to be 14.7 years, both for age of onset or exacerbation and for age at the moment of assessment. Finally, to better estimate the dispersion of age values, we also computed the standard deviations, which result in 2.66 for age of onset or exacerbation and 6.22 for age at the moment of assessment.

In total, we were able to retrieve thirty pertinent cases.

First, Park et al. [13] reported four cases of AN, with eating restrictions, which all developed shortly after a viral infection: three of them followed glandular fever (aged 19, 33 and 29), the fourth (35 years) ensued a pharyngitis with fever. However, no specific treatments are mentioned to have been performed to target the triggering infections, but only dietary treatment, in an outpatient or inpatient setting, clomipramine, or behavioral therapy. This study, in particular, referred to subjects older at the moment of assessment than those studied in the other reports, significantly contributing to the increase in the weight mean and standard deviation values, as mentioned above.

Another case study [14] reported three clinical cases. The first patient was a 12-year and 8-month-old boy who presented with symptoms of eating disorder, irritability, social withdrawal, sadness, and anxiety, and was hospitalized in an eating disorders unit. These symptoms had begun 10 months earlier than the study assessment, but a dramatic exacerbation happened 6 months later, after a 1-month precedent untreated upper respiratory tract infection. Two days after admission and after the amoxicillin administration, the patient’s behavior began to improve. One month after the hospital discharge, the anti-DNase B titer was 1:340 and the ASO titer was 400. The second case referred to a girl of 14 years and 9 months. She had contracted a severe Epstein–Barr virus infection at age 13. Antinuclear antibody titer was 1:320, whereas antistreptolysin O (ASO) titer, anti-DNase B, and throat culture were negative. She had experienced, at 13 years and 11 months, an exacerbation of the arthritis she suffered from, with a sudden increase in her eating disorder symptoms (a 40-pound weight loss in the next 4 months). Both the patient’s eating disorder and arthritis decreased with a treatment defined as “conventional”, not further specified. The third case was a 16-year-old boy with mild AN in remission at the time of evaluation. He had a series of upper respiratory tract infections 6 months earlier, and shortly after, obsessive symptoms and weight decrease became abruptly manifest. Anti-DNase B was moderately elevated at 1:340. Throat culture, ASO titer, and antinuclear antibodies were negative. The patient had a history of many upper respiratory tract infections in childhood, with many throat cultures positive for Group A Beta-Haemolytic Streptococcal, GABHS, even after antibiotic treatment. During the month after the study assessment, the patient’s eating disorder and obsessions progressively went into remission with no treatment needed.

A later study [3] reported four cases of children, who fulfilled at least four of the above mentioned criteria for PANDAS–AN, and were treated with antibiotics in an open trial and conventional therapies. One girl, aged 11 years, had suffered from a severe weight loss (at the age of 10 years and 6 months) associated with obsessive thoughts related to food and weight; 5 months earlier, she had suffered from a not properly treated sore throat associated with fever. Her anamnesis was mute, except for a history of upper respiratory tract infections. Psychometric tests were performed, including Children’s Yale–Brown Obsessive–Compulsive Scale, CY–BOCS [15], and The Yale–Brown–Cornell Eating Disorders Scale, YBC–EDS [16], which attested a severe grade for both eating disorder and obsessive symptoms. However, the patient fulfilled diagnostic criteria for AN, but not for OCD, since her obsessions and compulsions only referred to food and weight. The throat culture initially performed was negative to GABHS bacteria, anti-DNase B and ASO titers exceeded upper limits of normality, antinuclear antibody (ANA) titer was negative, the rate of positivity to D8/17 marker corresponded to the 31% of B lymphocytes. Generally, regarding the D8/17 marker, subjects with PANDAS–AN are more likely to be D8/17 + or to have a higher percentage of D8/17 + cells than those without any eating disorder or PANDAS [17]. After a high-dose therapy with amoxicillin, eating disorder and obsessive symptomatology gradually ceased.

Another case report referred to the clinical course of a girl, 12 years and 5 months, who had suddenly manifested severe eating restrictions (at age 11 years and 8 months), resulting in a severe weight loss, requiring multiple hospitalizations, accompanied by hyperacusis and slightly slurred speech. Two weeks earlier, she had suffered from a flu-like syndrome and fever. The medical history was positive to recurrent upper respiratory tract infections, periorbital headaches, nasal congestion; the psychiatric anamnesis was positive for OCD. An assessment of eating disorder and obsessive symptoms was performed on CY–BOCS and YBC–EDS tests, which certified the severity of symptomatology in both cases. The throat culture was positive for streptococcus, and anti-DNase B titer exceeded the upper limit of normality, whereas the ASO titer did not; the rate of positivity to D8/17 marker corresponded to the 28% of B lymphocytes. Concomitantly with the above-mentioned psychiatric symptoms, active sinusitis, headaches and nasal congestion were also observed. Even in this case, after an antibiotic treatment, both obsessive and eating disorder symptoms gradually disappeared.

The third case focused on a girl, 15 years, with a 4-year history of AN, binge-eating/purging subtype, in partial remission at the moment of the study assessment, with the persistence of clinical symptoms, such as eating restrictions, bingeing, purging and physical hyperactivity; furthermore, she had suffered from OCD, since she was 8. Differently from the previously cited cases, 1 year before the appraisal conducted in the work in analysis, she had suffered from streptococcal pharyngitis (positive throat culture), treated with antibiotics, after which eating disorder symptoms were reported to weaken. The medical history was positive to frequent bladder and upper respiratory tract infections, as well as headaches, nasal stuffiness, and amenorrhoea secondary to her eating disorder. Significantly, one maternal uncle had a history positive for a rheumatic fever at age 11, subsequently accompanied by the onset of obsessive symptomatology and muscle twitching. Psychometric evaluation was performed: scores on the CY–BOCS and YBC–EDS attested severity both in obsessive and eating disorder symptoms (respectively, 28 and 22). At the moment of assessment, the subject had an active sinusitis, and her throat culture was found positive for gram-negative rods. Anti-DNase B titer exceeded the upper limit of normality, ANA titer resulted as positive at 1:80, with a nucleolar pattern, while negative double-stranded DNA was negative; the rate of positivity to D8/17 marker amounted to the 38% of B lymphocytes. Some weeks later, a bilateral otitis media, associated with fever, added itself to the clinical complication. After multiple antibiotic therapies (ciprofloxacin was crucial), the infection was resolved, and an open trial of amoxicillin was started, which produced a progressive remission of OCD and eating disorder symptomatology.

The fourth case report referred to a young boy, aged 12 years and 8 months, who had suffered from AN (with sudden onset) for 10 months, with a subsequent acute worsening of eating disorder symptoms following an untreated severe upper respiratory tract infection, undergone by the patient many weeks before (age 12 years and 3 months). His medical history was positive for three brief periods of mild motor and vocal tics from age 3. Anti-DNase B and ANA titers, as well as throat culture, were negative, whereas ASO titer exceeded the upper limit of normality; the rate of positivity to D8/17 marker corresponded to the 35% of B lymphocytes. Clinical evaluation showed active sinusitis and mild choreiform movements when the case report was performed. Mild anxiety symptoms and hyperactivity/attentional problems were also present. Amoxicillin was administered, then suspended for a brief period, which resulted in further worsening of eating disorder symptoms. The recommencement of antibiotics, taken by the patient for 1 year more, produced a progressive remission both of obsessive and eating disorder symptoms, as showed by a decrease in CY–BOCS and YBC–EDS scores. Fluoxetine, at first, for the psychiatric symptoms, then sertraline, venlafaxine, and clonidine for the hyperactivity and attentional problems, were also administered.

Later, Sokol et al. [17] conducted another study aiming at investigating the prevalence of a suspected tract marker for PANDAS, known as D8/17, in 16 patients (15 girls and 1 boy, mean age 14.7, Standard Deviation, SD, 2.7) with possible PANDAS–AN. These subjects fulfilled DSM-IV criteria for AN, whose onset or exacerbation was acute, temporally related to antecedent or concomitant streptococcal infections (positivity for clinical history, throat culture, anti-deoxyribonuclease B titer and/or antistreptolysin O titer), prepubertal in 10 of them. 4 of these subjects had comorbid OCD, 11 subjects used one to three psychotropic medications (1 of them took levothyroxine), and 5 did not use any drugs.

Another case report was published [18], referring to an 8-year-old boy, with a 2-month history of significant weight loss, behavioral abnormalities, and recurrent culture-proven Group A Beta-Haemolytic Streptococcal (GABHS) pharyngitis. Group A Strep Direct antigen test, throat culture, blood culture, blood chemistry, anti-nuclear antibody, anti-streptolysin-O antibody titers, and Rheumatoid Factor were all reported as negative. At the same time, IgG levels amounted to 4.63, and levels of Anti-DNase B resulted elevated (1:960 on admission and 1:1360 after 3 weeks). CY–BOCS test was performed, certifying an extremely severe OCD (score: 33). Re-feeding was conducted via an ED protocol (including nasogastric, NG, tube), fluvoxamine was administered, later switched to sertraline (50 mg daily, then 25 mg); CBT and clarithromycin bis in die (prophylaxis prior tonsillectomy) were also performed.

Last, another study [19] examined the cases of 29 children, three of whom (10%) expressed concerns about weight or body shape and carried out a restrictive eating diet, experiencing a body weight decrement (in two of them the exact value was not available, while in the third the weight loss was of 0.9 kg). However, only two cases corresponded to the inclusion criteria adopted in the present study. Indeed, the first case referred to an 8-year-old girl with a history of exposure to GAS, and a successive development of restrictive eating (2 months long) accompanied by body image concerns and the feeling she did not deserve to eat or do pleasurable things. Flu-like symptoms and an increase in ASO and Anti-DNase B titers appeared. The girl also suffered from OCD. CY-BOCS and Yale Global Tic Severity Scale, YGTSS, scores, respectively, corresponded to 27 and 18. Amoxicillin (for 2 weeks), amoxicillin–clavulanic acid for GAS prophylaxis, and later IVIG were administered, with a full recovery and slight residual generalized anxiety. The second case referred to a 7-year-old girl, with a history of exposure to GAS and a successive development of restrictive eating (1 month long) accompanied by concerns about being overweight. CY–BOCS and YGTSS scores, respectively, corresponded to 34 and 18.

All cases are summarized in Table 2*.*

**Table 2:** Characteristics and data related to the studies

Authors, year of publication	Age and characteristics of onset or exacerbation of AN	Precedent infection/illness	Concomitant organic signs or symptoms	Other concomitant psychiatric symptoms	Psychometric tests performed	Therapy
Park et al., 1995 [13]	A 19-year-old girl, whose AN-onset occurred after an initial 5-kg weight-loss attributable to a glandular fever, with a subsequent sharp weight decrease, accompanied by a marked physical hyperactivity, requiring a hospital admission (30 kg, BMI 11.7 kg/m2). The admission period succeeded in producing a weight gain (up to 38 kg), but a rapid relapse occurred after discharge, resulting in a subsequent readmission	Glandular fever	Liver and cardiac enzymes raised	Depressive symptoms were noted by the general practitioner, alongside the progressive weight loss	Not reported	Dietary treatment (no other treatment specified)
Park et al., 1995 [13]	A 33-year-old woman with a 3-year history of AN, preceded by a 12-year history of subclinical eating disorder (with partial recovery and a rapid relapse), after a 12-kg weight loss caused by a glandular fever she had undergone at the age of 18. At the psychiatric assessment, she was found to suffer from the fear of getting fat, the feeling of guilt on eating, a severe body-image distortion; her weight was 35 kg (BMI: 12 kg/m2) after a 12-kg weight decrease	Glandular fever	A possible concomitant connective tissue disorder	Not reported	Not reported	A history of both outpatient and inpatient treatments
Park et al., 1995 [13]	A 29-year-old woman with a 13-year history of AN-restricting type, requiring multiple hospitalizations, whose onset took place shortly after a glandular fever-like illness driven by an adenoviral infection	A glandular fever-like illness driven by an adenoviral infection	Not reported	Not reported	Not reported	A history both of outpatient and inpatient treatments
Park et al., 1995 [13]	A 35-year-old girl, with a 20-year history of AN-restricting type, and a 6-year history of obsessive–compulsive disorder (OCD). Her eating disorder manifested shortly after a severe pharyngitis with fever, during which a 12-kg weight loss had occurred. AN-r symptoms soon developed, including physical hyperactivity and food restrictions, and the patient reached a minimum weight of 26 kg (BMI: 10 kg/m2) and underwent many hospital admissions	A severe pharyngitis with fever; investigations conducted were found as negative but did not include monospot or any other viral titers	Not reported	Not reported	Not reported	Temporary weight restoration, clomipramine and behavioral therapy were performed, the patient was referred for psychosurgery but resulted physically unfit for the operation and, at the moment of assessment, was waiting long-term care
Sokol et al., 1997 [14]	12-year and 8-month years old boy with a history lasting 10 months of eating disorder symptoms of abrupt onset dramatically increased 4 months before admission	An untreated upper respiratory tract infection 1 month before the severe exacerbation of symptoms, with high titers of anti-deoxyribonuclease B (1:340) and ASO (1:400), and negativity of antinuclear antibody titer and throat culture	On admission, sinusitis of moderate intensity and mild choreiform movements of all four extremities	Irritability, sadness, anxiety, and social withdrawal	Not Reported	Amoxicillin, after which the patient’s behavior began to improve
Sokol et al., 1997 [14]	14 years and 9 months years old girl, who went on a diet 8 months after a severe Epstein–Barr virus infection (contracted at age 13), concurrently developing obsessive concerns about eating and being overweight. When the patient was 13 years and 11 months, she suffered from an exacerbation of her arthritis, and shortly after her thoughts concerning eating and being overweight underwent an exacerbation and resulted in pathological eating behaviors	A severe Epstein–Barr virus infection at age 13, with positivity of antinuclear antibody titer (1:320), with a diffuse pattern and negativity of ASO titer, anti-DNase B, and throat culture	Postinfectious migratory polyarthritis at age 13 and 8 months	Not Reported	Not Reported	The patient’s eating disorder and arthritis decreased in parallel during the next 11 months with a treatment defined as “conventional”, not further specified. Both conditions went into remission shortly after the beginning of this evaluation, and no further treatment was needed
Sokol et al., 1997 [14]	16-year-old boy, who had, after some upper respiratory infections contracted 6 months earlier, a sudden onset of a mild form of AN, with obsessive symptoms related to food and an intense fear of weight increase	Some upper respiratory infections, with evidence of a moderate elevation of anti-DNase B at 1:340 and negativity of throat culture, ASO titer, and antinuclear antibodies	Obsessive symptoms	Not Reported	Not Reported	Five months after the infections were contracted, a progressive remission of eating disorder and obsessive symptoms was observed without the need for any treatment
Sokol 2000 [3]	One girl, aged 11 years, had suffered from a severe weight loss (at age 10 years and 6 months), associated with obsessive thoughts related to food and weight, fulfilling diagnostic criteria for AN-restricting subtype, but not for OCD; 5 months earlier, she had suffered from an upper respiratory infection (a sore throat), not properly treated, which associated with fever	Sore throat, not properly treated, which associated with fever, with negativity to Group A Beta-Haemolytic Streptococcal (GABHS) bacteria of the throat culture originally performed and of antinuclear antibody (ANA) titer, and positivity of anti-DNase B and ASO titers, the rate of positivity to D8/17 marker corresponded to the 31% of B lymphocytes	Sinusitis (also documented on MRI)	Not Reported	CY–BOCS and YBC–EDS, which attested a severe grade both of eating disorder and obsessive symptoms	After a high-dose therapy with amoxicillin, eating disorder and obsessive symptomatology gradually ceased
Sokol 2000 [3]	A girl, aged 12 years and 5 months, who had suddenly begun severe eating restrictions (at age 11 years and 8 months), resulting in severe weight loss, requiring multiple hospitalizations, after a flu-like syndrome with fever contracted 2 weeks before	Two weeks earlier she had suffered from a flu-like syndrome with fever, and positivity for streptococcus of throat culture, of anti-DNase B titer, and negativity of ASO titer, the rate of positivity to D8/17 marker corresponded to the 28% of B lymphocytes	Hyperacusis, slightly slurred speech, active sinusitis (also documented on MRI), headaches, and nasal congestion	OCD	CY–BOCS and YBC–EDS tests, which certified severity of symptomatology in both cases	After an amoxicillin- and cefuroxime-based treatment, both obsessive and eating disorder symptoms gradually disappeared
Sokol 2000 [3]	A girl, 15 years, with a 4-year history of AN, binge-eating/purging subtype, in partial remission at the moment of the study assessment, with the persistence of clinical symptoms, such as eating restrictions, bingeing, purging and physical hyperactivity. One year before the assessment conducted in this study, she suffered from streptococcal pharyngitis (positive throat culture), treated with antibiotics, after which eating disorder symptoms were reported to weaken	Streptococcal pharyngitis, with positivity for gram-negative rods of throat culture, of anti-DNase B titer, ANA titer (1:80), with a nucleolar pattern, and negativity of ASO and double-stranded DNA; the rate of positivity to D8/17 marker amounted to the 38% of B lymphocytes	At the moment of assessment, an active sinusitis. Some weeks after the streptococcal pharyngitis mentioned before, a bilateral otitis media, associated with fever, added itself to the clinical complication	Not Reported	CY–BOCS (score: 28) and YBC–EDS (score: 22)	After multiple antibiotic therapies (amoxicillin, sulfamethoxazole and, trimethoprim, ciprofloxacin, which was crucial), the infection was resolved, and an open trial of amoxicillin was started, which produced a progressive remission of OCD and eating disorder symptomatology
Sokol 2000 [3]	A boy aged 12 years and 8 months, who had suffered from AN (with a sudden onset) for 10 months, with a subsequent acute worsening of eating disorder symptoms following an untreated severe upper respiratory tract infection, undergone by the patient many weeks before (age 12 years and 3 months)	Severe upper respiratory tract infection, undergone by the patient many weeks before, with the negativity of anti-DNase B and ANA titers, of throat culture, and positivity of ASO titer; the rate of positivity to D8/17 marker corresponded to the 35% of B lymphocytes	Active sinusitis and mild choreiform movements when the case report was performed	Mild anxiety and hyperactivity/attentional problems	CY–BOCS and YBC–EDS scores were reported to decrease with the administration of antibiotics	Amoxicillin was administered, then suspended for a brief period, which resulted in further worsening of eating disorder symptoms. The recommencement of antibiotics, taken by the patient for 1 year more, produced a progressive remission both of obsessive and eating disorder symptoms, as showed by a decrease in CY–BOCS and YBC–EDS scores. Fluoxetine, at first, for the anxiety symptoms, then sertraline, venlafaxine, and clonidine, for the hyperactivity and attentional problems, were also administered
Sokol et al., 2002 [17]	16 subjects, 15 girls and 1 boy, with a mean age of 14.7 years (S.D. 2.7), fulfilling DSM-IV criteria for AN, whose onset or exacerbation was acute, temporally related to antecedent or concomitant streptococcal infections, prepubertal in 10 of them	Direct or indirect evidence of streptococcal infection (GAS), positivity for clinical history, throat culture, anti-deoxyribonuclease B titer and/or antistreptolysin O titer,	Minor neurological abnormalities	Four of the subjects with anorexia had comorbid OCD	Not reported	11 subjects used one to three psychotropic medications (1 of them took levothyroxine). Five did not use any drugs
Calkin and Carandang, 2007 [18]	A 8-year-old boy, with a 2-month history of significant weight loss, behavioral abnormalities, and recurrent culture-proven Group A Beta-Haemolytic Streptococcal (GABHS) pharyngitis	None reported, with negativity of Group A Strep Direct antigen test, throat culture, blood culture, blood chemistry, anti-nuclear antibody, anti-streptolysin-O antibody titers, Rheumatoid Factor, IgG (4.63 mg/mL), and elevated levels of Anti-DNase B (1:960 on admission and 1:1360 after 3 weeks)	Weight loss, behavioral abnormalities, history of Group A Beta-Haemolytic Streptococcal (GABHS) pharyngitis, choreoform-like running spurts, facial grimacing	Various tics and in anamnesis described as an anxious child; OCD	CY–BOCS (score: 33, certifying an extremely severe OCD)	Re-feeding via an ED protocol (including nasogastric, NG, tube), fluvoxamine later switched to sertraline (50 mg daily, than 25 mg), CBT, clarithromycin bis in die (prophylaxis prior tonsillectomy)
Toufexis et al., 2015 [19]	An 8-year-old girl with a history of exposure to GAS, and a successive development of restrictive eating (2 months long) accompanied by body image concerns and the feeling she did not deserve to eat or do pleasurable things	Exposure to GAS (rapid antigen detection reported as negative), with flu-like symptoms, increase in ASO and Anti-DNase B titers	None	OCD	CY–BOCS (score: 27) and YGTSS (score: 18)	Amoxicillin (for 2 weeks), amoxicillin–clavulanic acid for GAS prophylaxis, later IVIG, with a full recovery and slight residual generalized anxiety
Toufexis et al., 2015 [19]	A 7-year-old girl, with a history of exposure to GAS and a successive development of restrictive eating (1-month long) accompanied by concerns about being overweight	Exposure to GAS (rapid antigen detection reported as negative)	Not reported	Not reported	CY–BOCS (score: 34) and YGTSS (score: 18)	Not reported

*AN* Anorexia Nervosa; *BMI* Body Max Index; *ASO* antistreptolysin O; *CY-BOCS* Children’s Yale–Brown Obsessive–Compulsive Scale; *YGTSS* Yale Global Tic Severity Scale; *GAS* Group A streptococcusl; *IVIG* IntraVenous ImmunoGlobuline; *GABHS* Group A Beta-Hemolytic Streptococci; *OCD* Obsessive–Compulsive Disorder; *SD* standard deviation; *DSM* Diagnostic and Statistical Manual of Mental Disorders; *YBC–EDS* Yale–Brown–Cornell Eating Disorders Scale; *ANA* Antinuclear Antibody; *NG* nasogastric; *CBT* Cognitive Behavioral Therapy; *ED* Eating Disordes

## The case

V. is a 17-year-old girl who started exhibiting eating disorder symptoms, later recognized as diagnostic of AN, restricting subtype, right after an intestinal infection. She arrived at our Eating Disorder Unit on 21 February 2024. Written informed consent for the publication of this study was obtained both from the girl and her parents.

### Socio-relational anamnesis

V. cohabits with her mother and two minor twin siblings, aged 15; her parents are separate, and a good relationship is reported between them. V. has good academic achievements. She had been practicing contemporary dance until June 2023, when she stopped due to difficulties related to keeping up with school schedules. V. referred to benefit from a strong circle of friends. Her menarche dates to July 2020, and she was on secondary amenorrhoea from September 2023 to July 2024. At the moment, her menstrual flow is reported as regular.

### Past medical history

The patient was born at 38 weeks via a caesarean section (birth weight: 2870 g). In the first 3 years of life, pediatric visits attested a good health status. V. was breastfed until the fourth month of life, when she started a mixed feeding; at the 8th-month assessment, the interruption of breastfeeding was reported. Her diet was described as diverse from 8 months until 3 years of life, when at the 3/4-year assessment, her nutrition was described as poor; however, at the 5/6-year assessment, her diet was re-evaluated as diverse. At 8 years and 10 months, the patient weighed 27.5 kg (50° percentile).

Furthermore, at the age of 6/7 years, the patient underwent further hospitalizations and accesses to the emergency department. In the first episode, the patient was admitted due to a 15-day lasting abdominal pain associated with diarrhoea and vomiting, in absence of fever, probably attributable to intestinal dysbiosis. The patient was also diagnosed with bronchitis. Later, she accessed twice again the emergency department due to a persistent abdominal pain associated, in the first case, with a bronchitis. Later, V. made another access to the emergency department due to persistent pain in the right side of abdomen. Both the patient and her parents considered these multiple episodes of abdominal pain to be possibly linked to stress, since they co-occurred with a period (2013–2014) of problems in the relationship between the parents, which culminated in their separation.

The medical and psychiatric history of V.’s parents was reported as mute, except for arterial hypertension in the father.

### Past psychiatric history

The psychiatric history of V. was reported as mute, except for a period of depressed mood during middle school due to bullying from which she reported to have suffered. Furthermore, the above-mentioned episodes of possibly psychosomatic abdominal pain deserve mention.

### Recent medical and psychiatric history

In August 2023, the patient, in good health, weighed 46 kg (32° percentile for height and 9° of weight; BMI: 17.97 kg/m^2^). In September 2023, the patient suffered from an intestinal infection, with abdominal pain, nausea, vomiting, and diarrhea, which lasted for about a week and was treated with probiotics, with only partial benefit. Some days after the resolution of the infection, which had resulted into weight loss, V. started severe quantitative and qualitative eating restrictions to lose further weight, even due to a sedentary lifestyle, that she was convinced to be conducting after stopping the sports activity. Her last menstrual flow was in early September. The patient referred not to have ever experienced a similar symptomatology, even though she had always been attentive to her physical appearance and body weight. The patient had a visit with a dietician, who suggested some dietary supplements and vitamins, which she managed to assume only partially. V. kept reducing her dietary intake, and her body weight gradually decreased. The patient referred a 7 kg-weight loss from December 2023 to January 2024. She denied any compensatory behaviors. Furthermore, concomitantly with abnormal eating behaviors, V. also manifested mood depression, irritability, anxiety, sleep reduction (few hours a night, with multiple awakenings), and some attention deficits. V. also suffered from organic symptoms, such as joint pain, especially in the knees, at night and during exertion (e.g., when the patient climbed stairs), night sweats, blurred vision, retro auricular eczema, and itch.

At the end of February, V. attended a visit to the outpatient service of our Eating Disorder Unit, when a diagnosis of AN, restricting subtype**,** was formulated by a psychiatrist expert in the field of ED treatment. Due to the severity of the illness, V. accessed the emergency department of the hospital and was admitted to the inpatient ward of our Eating Disorder Unit.

### During the hospital admission

On February 21st, her body weight was 34.5 kg, 160 cm, BMI: 13.47 kg/m^2^ (height 160 cm) (3° percentile of weight**)**. The weight loss was of 11.5 kg (25% of body weight lost in 5 months).

The patient was hospitalized in our Eating Disorder Unit for 29 days (from 21/2/24 to 20/3/24). At admission, the psychiatric examination noted body dysperception, recurrent thoughts and obsessive ruminations concerning eating themes, depressed mood and a sleep–wake rhythm poorly regulated by medicines.

During the hospitalization, the following blood tests were altered: at the admission s-25-OH Vitamin D 23.6 ng/mL; folic acid 13.8 ng/mL; B12 vitamin 1121 pg/mL; ferritin 288 ng/mL; Na 132 mmol/L; Cl 96 mmol/L; Ca 0.95 mmol/L; HDL cholesterol 80 mg/dL; total cholesterol 206 mg/dL; total bilirubin 3.7 mg/dL. At the end of the hospitalization all the exams were in range, except for a mild anaemia (Hb 11.1 g/dL) (Table 3).

**Table 3:** Test results during hospitalization

Abnormal values—admission	Abnormal values—discharge
B12 vitamin: 1121 pg/mL; Folic acid: 13.8 ng/mL; S-25-OH Vitamin D: 23.6 ng/mL; Ferritin: 288 ng/mL; Na: 132 mmol/L; Cl: 96 mmol/L; Ca: 0.95 mmol/L; HDL cholesterol: 80 mg/dL; Total cholesterol: 206 mg/dL; Total bilirubin: 3.7 mg/dL	Hb: 11.1 g/dL

*S-25-OH* serum-25-hydroxivitamin; *Na* sodium; *Cl* chloride; *Ca* calcium; *HDL* High Density Lipoprotein; *Hb* haemoglobin

During the hospitalization some tests were performed to confirm the PANS suspect, and the values were: s-Ab anti phospholipase A2 receptor (ELISA): <1/ μL (negative), ASLO 265 UI (positive) and anti-DNase B 230 UI (positive); the WBC were 5790/μL at the admission and 6170/μL at the moment of the discharge, while the PCR was 0.2 mg/dL at the beginning and stood stable, while the patient was hospitalized.

During the admission period, moreover, were performed dietetic and dietary visits, bio-impedance spectroscopies and multiple psychiatric visits with daily visits with the doctors.

Based on the clinical history and previous infection, were also performed: dermatological investigation, rheumatological visit, bone densitometry, echocardiography, urine culture, and throat swab. All these exams showed no pathological signs.

From the nutritional point of view, a minimum scheme (800 kcal) was initially set and afterwards progressively increased until the discharge caloric intake (1200 kcal) was reached, partly introduced via a nasogastric tube.

At the discharge, the therapy was set with Sertraline 75 mg, Olanzapine 2.5 mg, 1 tablet, Alprazolam 0.75 mg/ml 20 drops, Lorazepam 1 mg, as needed, before meals, cetirizine, as needed, and food and vitamin supplements.

Psychometric tests were administered: the Structured Clinical Interview for DSM disorder, fifth edition, SCID-5, which suggested possible OCD symptoms in the patient; the YBC–EDS, which attested moderate eating-related obsessive symptomatology (total score: 19).

The patient underwent rehabilitation treatment in a residential care for the subsequent months. After 2 months of rehabilitation, the patient reached the value of 39.5 kg, height 160 cm, BMI: 15.4 kg/m^2^. The increase, up to that time, approximately amounted to 4 kilos. The mental status examination noted a still partially depressed mood and a sleep–wake rhythm regulated by medicines.

At the patient’s discharge, in June, the 17th, the mental status examination noted less severe body dysperception, thinking less polarized on the eating theme, still partially depressed mood and a sleep–wake rhythm regulated by medicines, preserved. Subsequently, V. has continued her treatment as an outpatient at the CER DCA U (University of Turin—AOU Città della Salute Hospital). After a month, the mental status examination resulted globally normal, except for a mild body dysperception and a mild polarization of thinking on the eating theme, and her anthropometric parameters were: 44.8 kg, height 160 cm, BMI: 17.5 kg/m^2^; the YBC–EDS was administered, and attested mild eating-related obsessive symptomatology (total score: 15), which corroborated the global clinical impression of an improvement in the patient status. The last outpatient follow-up visit, conducted 3 months after discharge from a residential care facility, confirmed both the trend of weight gain (50 kg, BMI 19.5 kg/m^2^) and a further reduction in obsessive thoughts related to food.

## Conclusions and discussion

Although there are promising hints and a theoretical model supporting a possible inflammatory pathogenesis underlying the development of some cases of AN [1, 12], the present literature review has led to insufficient and contradictory evidence. In the past, the nosological construct of PANS/PANDAS was also proposed for AN, but in about 20 years, only 30 cases have been described in the literature. Furthermore, all the cases described are reported as single case reports or case series. From the detailed analysis of the clinical pictures, some common factors are found in most of the cases described—namely, abrupt onset, development of psychiatric comorbidity [20], positivity to serological tests for recent or concomitant infections—but the published studies show a wide variability of clinical presentation, and both psychometric and serological evaluation. In fact, in some cases, the infection is present, in others it is not; in some cases, the onset is without any previous symptoms, in other cases, the infection seems to exacerbate the symptoms already present; in some cases there is a clear recent history of infection, in other cases the history is less clear. Furthermore, each case was treated with the usual treatment for AN, only sometimes with adjunctive antibiotic therapy. The observation of a positive response not only to the infection but also to the eating disorder-related symptoms would hold substantial significance. This finding may indicate a partially shared etiopathogenetic pathway underlying both the infection and the eating disorder, whether triggered or exacerbated by it. Further studies in this field may help in developing a unified pharmacological approach.

Therefore, it is difficult to understand whether the subjects are simply rapid responders or, once the infection is resolved, the eating symptoms dissolve accordingly.

To date, therefore, there is no robust data to draw any conclusions on the possibility that previous infections can trigger AN. Furthermore, although psychoneuroendocrinoimmunological theories hypothesize a cause and effect relationship [10] and although there is still an increased risk of developing a mental disorder after infection [9], the coexistence of AN and concomitant infections may be coincidental. Despite these limitations, it would be helpful to study this research area better and more thoroughly for at least three reasons. First, genetic evidence is starting to support a possible weak link between autoimmune diseases and mental disorders [21], including AN [22]. Second, interest is growing in studies on the association between autoimmunity and neurodevelopmental disorders [23] and between autoimmunity and mental disorders. Third, the debate on long-COVID-19 has recently drawn attention to neuroimmunology [24] with evidence from experimental models [25] and contradictory data from clinical research [26], with the involvement also of psychological reaction mechanisms to infectious diseases.

One of the significant reasons for the failure of the actual research is the predominance of studies that include childhood or early adolescence cases. This cutoff restricts the in-depth analysis of this topic too much. If it is true that the age of onset is decreasing, most cases begin after the age of 14, with a peak around 15.5 years [5]. For this reason, we reported a case of a young girl. The present case report seems to suggest a correlation between the acute development of the restrictive symptoms experienced by the patient and the previous infection. Even the rapid and positive course, which is not usual in EDs [27], may suggest a peculiar and non-typical clinical picture. Finally—however, non-specific—PANS criteria are overall met by the patient, since she also developed abrupt neuropsychiatric symptoms, such as mood depression, irritability, anxiety, sleep reduction, and some attention deficits. On the other side, we did not retrieve in past literature any other neuropsychiatric syndrome similarly triggered by an intestinal infection. Indeed, glandular fevers, pharyngitis with fever, respiratory infections, GABHS-driven sore throats, and flu-like syndromes have been described as possible infectious antecedents of subsequent abrupt onset or exacerbation of eating restrictions [3, 13, 14, 17–19]. Thus, the present case can be considered along the spectrum of post-infectious manifestations of AN or restrictive eating but with an atypical and unprecedented trigger. Another peculiarity is the girl’s late-adolescent age, compared with the childhood age usually considered in literature. Past studies mainly focused on cases of children and adolescents, with a weighted mean age of 13.86 years and some very young cases under 10 years (as concerns those analyzed in the present mini-review). Thus, this atypical manifestation of PANS might, at the same time, raise interest in its novelty. Still, it also suggests the need for further studies that could better clarify this correlation.

However, the body of literature on the theme of acute-onset post-infectious AN strongly suggests that infectious disease history should always be evaluated in relation to eating disorder symptoms, also in light of the growing interest in the complex link between immune-mediated diseases and the development of psychiatric symptoms [28] and the need to improve the research in the EDs spectrum [29]. Thus, research would benefit from more laboratories willing to conduct secondary analyses, which in our case was only partly possible. These challenges, related to the diagnostic path, have been pointed out as a critical issue even in a recent review [30], which underlines the importance of a standardized diagnostic approach. According to these authors, it should be based on the investigation of non-specific markers of inflammation, such as ESR or PCR, specific markers of streptococcal infections (ASLO, anti-DNase B), and specific markers of autoimmune reactions (antineuronal antibodies, D8/D17). The last, regarded by recent literature as crucial, challenged us the most because of difficulties in executing them.

In conclusion, this topic remains complex and raises alternative scenarios: not only have infectious diseases been found to be possible triggers of autoimmune processes, which can lead to AN or other restrictive behaviors, but also, without any autoimmune mechanism, infections could lead subjects to a first experience of weight loss, subsequently triggering the onset and maintenance (especially on a psychological level) of eating restrictions. In any case, infections are expected to be increasingly recognized as relevant factors in the development and worsening of eating restrictions and should always be considered in the diagnostic assessment of recent cases of AN, especially in younger patients.

## Strengths and limits

The present study not only consists of the first review on the theme of abrupt onset or exacerbation of AN following recent infections but also provides the report of a well-conducted case whose course was novel in its manifestations and where tests and laboratory examinations were accurately administered and periodic checks during the hospitalization and the follow-up period after the patient’s discharge were attentively performed.

However, this review relies mainly on case-series and, more generally, on the few publications available on a highly debated theme that still raises unsolved questions. Moreover, a minor portion of cases included in this review exhibited eating behaviors that did not fulfill criteria for a full diagnosis of AN, according to DSM-5-TR [4], but in some instances displayed features resembling those of atypical AN. Furthermore, the atypicality of our case report might raise doubts about its actual post-infectious nature, and, in any case, we did not have the opportunity to perform all the diagnostic tests recommended for formulating an exact diagnosis of PANS/PANDAS.

### What is already known on this subject?

A discrete body of literature exists on pediatric acute-onset neuropsychiatric syndromes, typically ensuing an infective event, that include severe food restrictions or obsessive–compulsive disorder, with the underlying hypothesis of a link between autoimmunity and AN.

### What this study adds?

The novelty of this study is that it offers a review, the first, as far as we know, of the cases available in the literature of abrupt onset or exacerbation of AN following recent infections. Moreover, the case report included in the study is interesting because of its rapid and favorable course, which is uncommon in the classical manifestations of AN. Furthermore, it offers insights into an atypical PANS presentation as a possible neuropsychiatric sequela of intestinal infection and as a late-onset syndrome when compared with the median onset age of the other studies we analysed in the present review.

## Data Availability

No datasets were generated or analysed during the current study.
